# Impact of a 15-month multi-channel continuous distribution pilot on ITN ownership and access in Eastern Region, Ghana

**DOI:** 10.1186/s12936-018-2275-8

**Published:** 2018-03-22

**Authors:** Celine Zegers de Beyl, Angela Acosta, April Monroe, Felix Nyanor-Fosu, Joshua Kweku Ofori, Obed Asamoah, Prince Owusu, Sureyya Hornston, Lilia Gerberg, Megan Fotheringham, Albert Kilian, Hannah Koenker

**Affiliations:** 1 USAID NetWorks Project, Malaria Consortium, London, UK; 2 USAID NetWorks and VectorWorks Projects, Johns Hopkins Bloomberg School of Public Health, Center for Communication Programs, Baltimore, MD USA; 3 USAID NetWorks and VectorWorks Projects, Johns Hopkins Bloomberg School of Public Health, Center for Communication Programs, Accra, Ghana; 4 USAID NetWorks Project, Malaria Consortium, Accra, Ghana; 5 President’s Malaria Initiative (PMI) - Ghana, United States Agency for International Development, Accra, Ghana; 6 U.S. President’s Malaria Initiative, U.S. Agency for International Development, Bureau for Global Health, Office of Infectious Disease, Arlington, VA USA; 7 USAID NetWorks and VectorWorks Projects, Tropical Health LLP, Montagut, Spain

**Keywords:** ITN, ITN distribution, Continuous distribution, School distribution, ANC distribution, EPI distribution, Malaria

## Abstract

**Background:**

Insecticide-treated nets are a key intervention for malaria prevention. While mass distribution can rapidly scale up ITN coverage, multiple channels may be needed to sustain high levels of ITN access and ownership. In Ghana’s Eastern Region, a continuous ITN distribution pilot, started in October 2012, 18–24 months after a mass campaign. The pilot distributed ITNs through antenatal care services (ANC), child welfare clinic services (CWC) through the Expanded Programme on Immunization, and to students in two classes of primary schools.

**Methods:**

ITN ownership and access were evaluated through two cross-sectional surveys, conducted at baseline in April 2012, 11–15 months after the mass campaign, and at endline in December 2013, after 1 year of continuous distribution. A representative sample was obtained using a multi-stage cluster sampling design. Household heads were interviewed using a structured questionnaire.

**Results:**

Household ownership of at least one ITN was 91.3% (95% CI 88.8–93.9) at baseline and was not statistically significant at endline 18 months later at 88.3% (95% CI 84.9–91.0) (p = 0.10). Ownership of at least 1 ITN per two people significantly decreased from 51.3% (95% CI 47.1–55.4) to 40.2% (95% CI 36.4–44.6) (p < 0.01). Population access to an ITN within the household also significantly decreased from 74.5% (95% CI 71.2–77.7) at baseline to 66.4% (95% CI 62.9–69.9) at endline (p < 0.01). The concentration index score for any CD channel was slightly positive (0.10; 95% CI 0.04–0.15).

**Conclusion:**

Thirty-one months after the mass campaign, the 15 months of continuous distribution activities had maintained levels of household ownership at least one ITN, but household ownership of one ITN for every two people and population access to ITN had declined. Ownership and access were higher with the CD programme than without. However, the number of ITNs delivered via ANC, CWC and two primary school classes were insufficient to sustain coverage targets. Future programmes should implement continuous distribution strategies fully within 1 year after a campaign or widen eligibility criteria (such as increase the number of classes) during the first year of implementation to make up for programme delays.

## Background

Insecticide-treated nets (ITN) are an important tool for preventing malaria-related morbidity and mortality, estimated to be responsible for 68% of the over 600 million cases averted since 2000 [1]. Most ITNs have been distributed through mass campaigns, which have been shown to increase coverage rapidly and equitably [2–14]. However, births, migration, and ITN damage erode coverage gains and require repeated or ongoing inputs of new ITNs. Recognizing the importance of sustaining universal coverage, the World Health Organization (WHO) began recommending a combination of mass campaigns and continuous distribution (CD) channels in 2013 to reach ownership and access targets [15].

Recent pilots have shown that programme areas with continuous distribution through community agents (Madagascar and South Sudan) or schools (Tanzania) are more likely to have sustained or higher ITN access levels than without the pilot approximately 2 years after a mass campaign [16–18]. However, these studies examined the contribution of community and school channels alone, without the implementation of health facility-based distribution through antenatal care (ANC) or the Expanded Programme on Immunization (EPI), a common policy in many sub-Saharan countries. To understand which combination of channels are appropriate for efficiently maintaining coverage levels, a multi-channel, continuous distribution model was tested in Ghana shortly after a mass campaign.

Malaria contributes significantly to morbidity and mortality in Ghana, with 24.2 million people at risk of the disease. National parasite prevalence in children under five has remained stable at around 27–28% between 2011 and 2014, and while outpatient department cases increased significantly due to National Health Insurance expansion and improved access to health care, institutional case fatality rates for children under five have dropped from 14.1% in 2000 to 0.6% in 2012 [19]. In 2010–2012, Ghana implemented a national mass campaign with a goal of universal coverage. During this period, 12.5 million ITNs were distributed nationwide through a door-to-door ‘hang-up’ strategy [20].

In November 2011, implementing partners and the National Malaria Control Programme developed a draft strategy to maintain ITN ownership and access after the campaign. NetCALC 1.0, an open-access population-based modelling tool, was used to select the combination of channels and the number of classes for the school distribution, with estimates based on targets set at maintaining 90% of households owning at least 1 ITN. The major components of the strategy were ITN distribution to: (1) pregnant women through ANC, (2) infants through measles II booster visits at Child Welfare Clinics (CWC), (3) children enrolled in class 2 and 6 of public and private primary schools. Three additional components of the strategy were expected to reach (4) children aged 4 during measles II booster ‘mop up’ campaigns (estimated at 2.8% of the total population), (5) secondary school students purchasing ITNs as part of school supply lists for boarding schools (estimated to reach 50% of one class of secondary school students per year), and (6) an estimated 10% of households purchasing one ITN per year through commercial channels. NetCALC models showed that these six channels would, in theory, be able to maintain universal coverage in Ghana if implementation began before the universal coverage campaign nets begin to wear out. Eastern Region was the first region to be fully covered in the mass campaign, in two phases in November–December 2010 and in April–May 2011, and was therefore selected as the pilot region.

## Methods

### Programme implementation

**Fig. 1:**
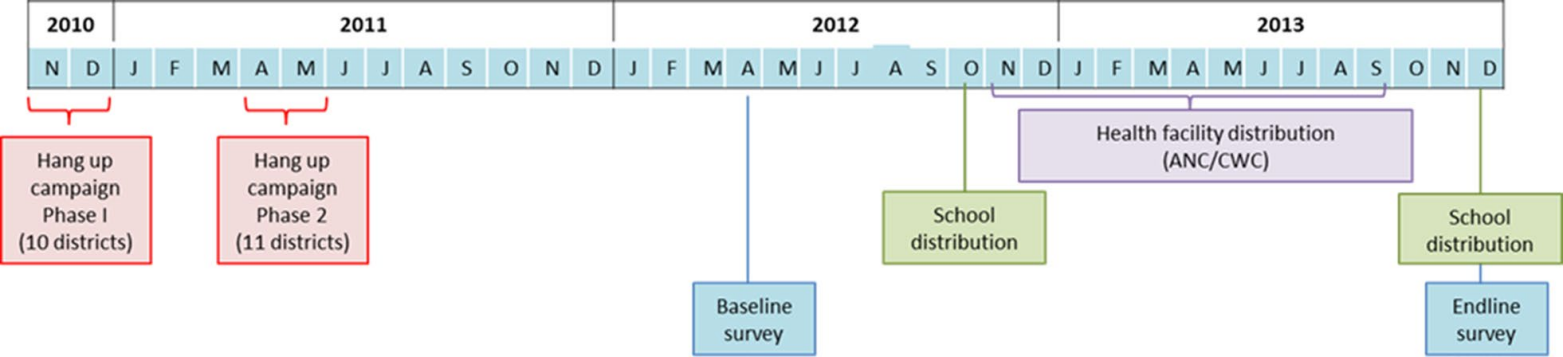
Timeline of study and intervention activities in Eastern Region, 2010–2013

Although six channels had been envisaged, only three channels were implemented during the pilot period. Students in primary class 2 and 6 received ITNs in October 2012. In November 2013, primary class 4 and 5 received ITNs, shown in Fig. 1. This change in classes was made because nation-wide school distribution was expected in May 2014, toward the end of the school year, serving classes 2 and 6; planners wanted to avoid distributing ITNs twice in a single school year to the same classes. The health facility and school distributions used existing structures within ANC and CWC clinics and schools, namely the storage, records, beneficiaries, and staff. Implementation required coordination between three main government agencies, the Ghana Health Service’s NMCP, the division of Reproductive and Child Health, the division of EPI, and the Ghana Education Service’s GES School Health Education Programme (SHEP). Representatives from each agency were involved in planning, execution and supervision at all levels: national, regional, districts, circuits/sub-districts and school or health facility level.

The number of ITNs distributed through schools was quantified using student enrolment data for the target classes. Twenty-six district SHEP coordinators and 180 circuit supervisors were trained on how to collect and compile enrollment data from schools and how to distribute ITNs and complete reports. ITNs were transported to district education stores and then directly to schools by circuit supervisors. Radio messaging and dramas accompanied school distributions to promote awareness, answer questions about eligibility, and encourage ITN use.

For the final 10 months of the pilot period, there was an increased focus on improving ITN distribution through health facilities through the provision of training, supervision visits and ITNs. All public and private health facilities that offered ANC and CWC services in Eastern Region participated in the programme. Every pregnant woman who visited ANC for the first time was entitled to receive an ITN, and given information about the effects of malaria in pregnancy and the need for proper use of nets. At CWC clinics, every child aged 18–24 months receiving measles II booster dose was entitled to an ITN. The choice to provide the ITN at the measles booster was made partly to boost rates for this new vaccination. To facilitate record-keeping, the Maternal Record Book and Child Health Card was modified to include “ITN given” and ITNs issued were recorded in ANC and CWC registers.

During the study period, 135,070 ITNs were distributed in the first round of school distribution in October 2012, and 136,000 were distributed in the second round in November 2013. Students in 2682 public and private primary schools received ITNs. During the same period, 114,000 ITNs were allocated across all health facilities in the 26 districts. All told, 385,070 ITNs were distributed during the pilot. NetCALC modelling indicated that this would have been sufficient to maintain levels of 90% household ownership of at least 1 ITN, and 77% population ITN access for 2012 and 2013, on the assumption that the Eastern Region campaign had achieved those coverage targets in 2011 based on distribution data.

### Survey design

A baseline survey was conducted in April 2012, 12–16 months after the mass campaign, and about 4 months before the start of CD activities (Fig. 1). The endline survey was carried out in December 2013, after 1 year of CD implementation, including two rounds of school distribution and 10 months of health facility distribution. Ghana has two rainy seasons each year, from April to July and from September to November; baseline data collection took place at the beginning of the first rainy season, while the endline data collection fielded near the end of the second rainy season. The primary outcomes of interest were ownership of at least one ITN and population access to an ITN, as defined by RBM’s Malaria Evaluation Reference Group [21]. Secondary outcomes included levels of over and under-supply within households, the relative contribution of each channel to ITN ownership, and ITN use.

### Study population

A multi-stage cluster sampling design was used in both surveys to ensure comparison across time. A cluster was defined as a community and 60 clusters were selected using systematic sampling with probability proportionate to size (PPS) based on population data from the campaign’s household registration lists. Within each cluster, a list of households was prepared by the survey team and households were then randomly selected for interview. If a cluster had more than 200 households, an equal-size section approach was used and one section was randomly chosen from the household list. Households were defined as “people eating from the same pot” which was the definition used in the mass campaign. Seventeen households per cluster at baseline and fifteen households per cluster at endline were targeted. To demonstrate that household ownership of 1 ITN for every 2 people was maintained between baseline and endline surveys, the standard formula for an equivalence study was used [22]. Sample size was calculated using an alpha error of 95%, a beta error of 80%, a design effect of 1.75, an anticipated non-response rate of 5%, and the expectation that there would be 5.0 persons per household, 15% of the population under 5, 4% of the proportion was pregnant, and that the percentage of households with 1 ITN for two people would be 49.8% at baseline and 48% at endline. The population estimates were based on the 2008 Ghana Demographic and Health Survey [23].

### Data collection and analysis

For data collection, the same pre-tested questionnaire was used for baseline and endline data collection. The primary respondent was the head of household or his/her spouse and the person who was present during the visit of mass campaign team. The questionnaire was based on the Malaria Indicator Survey and focused on household ownership and use of ITNs. Questions were added to capture several processes specific to continuous distribution such as the number of ITNs received through school, ANC, or CWC as well as the number of eligible students within the household. ANC/EPI (CWC) nets from the pilot were nets reported from those sources that were also obtained during the pilot period, as determined the question on “how many months ago did you obtain this net”. Double entry of all records was done using EpiData software version 3.1. Both data sets were then compared and any discrepant record was verified from the original questionnaires. Data were then transferred to Stata 14.0 statistical software package for further consistency checks and preparation for analysis. All analysis was done adjusting for the cluster sampling by using the “svy” command family in Stata. Concentration index and concentration curves were used to analyse outcome differences by wealth. Standard errors and confidence intervals for the concentration indices were calculated using the formula suggested by Kakwani et al. [24].

## Results

### Sample

The baseline survey included 1014 households (99.4% of the target), with 5226 household members. Nearly all (99.0%) of them were usual residents (de jure population) and 96.7% stayed in the house the previous night (de facto population). The baseline sample included 2336 nets, of which 2278 (97.5%) were ITNs. The endline survey included 898 households (99.8% of the target), 1865 nets (98% ITNs) and 4862 household members. Most household members (98.4%) were usual residents and 95.6% had stayed in the house the previous night.

Table 1 shows household characteristics at baseline and endline. Households were similar in terms of household composition, urban/rural residence and highest level of education achieved by the head of household. However, households from the endline survey were slightly more likely to have a child under five and slightly more household members. The percent of households with a tin roof and a mobile phone was somewhat higher at baseline, suggesting greater wealth. Lastly, households from the endline survey were less likely to report having received ITNs from the 2011 campaign; those who did also reported receiving fewer nets.

**Table 1:** Characteristics of surveyed households and population and their participation in the ITN distribution campaign at baseline and endline

Characteristics	Baseline (N = 1014)	Endline (N = 898)	p value
Mean # of de-jure (usual) household members	5.15	5.41	0.06
Mean # of persons per sleeping room	2.54	2.55	0.9
Households with a child under five in %	41.9	46.6	0.02
Households with a pregnant woman in %	5.8	3.9	0.09
Households headed by females in %	35.0	33.7	0.6
Mean age of head of household in years	50.4	51.3	0.5
Highest education level attained by head of household in %			0.1
No schooling	24.9	25.7	
Primary	32.3	37.2	
Secondary	33.6	30.5	
Higher than secondary	9.2	6.6	
Urban residence in %	38.2	44.9	0.3
Houses with modern roof (tin or zinc) in %	90.1	94.0	< 0.01
Household ownership of mobile phone in %	81.2	86.6	0.01

### ITN ownership, access and use

**Fig. 2:**
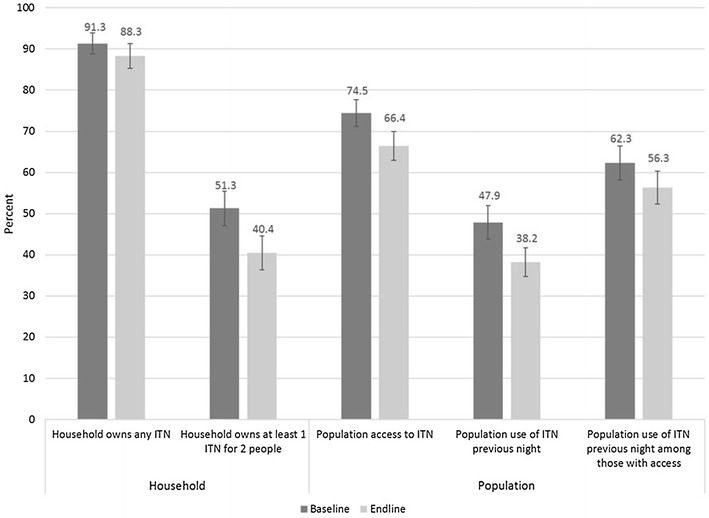
ITN Ownership, access, and use at baseline and endline

Levels of ITN ownership, access and use between baseline and endline are compared in Fig. 2. Ownership of at least one ITN remained stable, with no statistical difference between baseline (91.3%; 95% CI 88.8–93.9) and endline (88.3%; 95% CI 84.9–91.0) (p = 0.10). Ownership of at least 1 ITN per two people significantly decreased from 51.3% (95% CI 47.1–55.4) to 40.2% (95% CI 36.4–44.6) (p < 0.01), as did population access to an ITN from 74.5% (95% CI 71.2–77.7) to 66.4% (95% CI 62.8–69.9) (p < 0.01). Household supply levels also declined, with the proportion of households with insufficient nets (ranging from zero to 1 ITN/3 people) increasing from 48.8% at baseline to 59.6% at endline (p < 0.01) (Table 2).

**Table 2:** Household ITN supply at baseline and endline

	Baseline (N = 1014)	Endline (N = 898)	p value
At household level			
ITN supply in %			< 0.01
No ITNs	8.7	11.6	
Less than 1 ITN/3 people	16.9	23.5	
1 ITN/3 people	23.2	24.5	
1 ITN/2 people	39.0	32.4	
1 ITN/person or more	12.3	8.0	

**Fig. 3:**
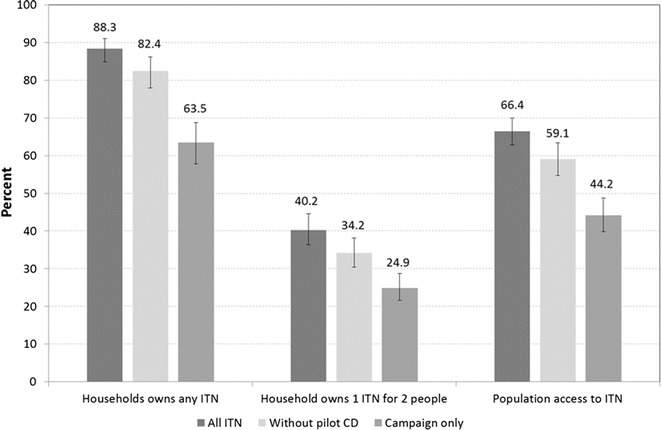
Ownership and access to ITNs at endline, calculated with all ITNs, excluding school and health facility ITNs, and excluding all ITNs except for 2010–2011 mass campaign ITNs. Nets reported received from the campaign, but whose age was less than 14 months, were coded as ‘unknown’

To assess the contribution of the continuous distribution channels to overall coverage, additional ITN ownership and access indicators were calculated at endline. Figure 3 presents key indicators for all ITNs in the sample (dark gray), all ITNs except those reported obtained from schools or health facilities (light gray), and for only ITNs from the 2010–2011 mass campaign (medium gray). These counterfactuals approximate what ITN ownership and access would have been if there had been no school or health facility distribution, or no distribution since the 2010–2011 mass campaign. There was no statistical difference in coverage indicators when ITNs coded as from school and health facilities were excluded (Fig. 3, light gray bars). However, ownership of 1 ITN for 2 people and population access to ITNs were both statistically significantly lower at endline when excluding all ITNs (health facility, schools, unknown, etc.) except those received from the 2010–2011 campaign (Fig. 3, medium gray bars).

### Source of ITNs

Table 3 shows the reach of various sources of ITNs. At baseline, almost all households had obtained at least 1 ITN from the recent or previous campaign (91.1%). The private sector was a distant second source (6.1%). Very few households owned ITNs from ANC (1.6%), or child health services (0.6%), and none from schools. Other sources such as the private sector or family/friends were also minimal. By the endline, however, the reach of CD channels had increased. Over 60% of households still owned an ITN from the campaigns, but 13.8% of households owned at least one ITN from schools, 8.0% owned a net from ANC, and 5.3% owned a net from CWC. Households owning any ITNs from the private sector remained stable at around 4%, while households owning nets from other sources (including family/friends) had increased to 7.3%. Nearly a quarter (23.8%) of households owned at least 1 ITN from any of the CD channels. The proportion of nets from unknown sources increased from 0.6% at baseline to 13.5% at endline.

**Table 3:** Percent of households with at least one ITN from various sources

Percent of households who own at least one ITN from:	Baseline (n = 1014)	Endline (n = 898)	p value
Campaign of 2010/11	89.6	63.5	< 0.001
ANC	1.6	8.0	< 0.001
EPI (CWC)	0.6	5.3	< 0.001
ANC/EPI (CWC)	2.2	12.5	< 0.001
ANC/EPI (CWC) only pilot		7.7	
Primary school	0.0	13.8	< 0.001
Any CD channel	2.2	23.8	< 0.001
Any CD channel (only pilot)		20.4	
Private sector	3.9	4.6	0.5
Other (family, friends, NGO)	3.3	7.3	< 0.001
Unknown	0.6	13.5	< 0.01

ITNs reported received from the campaign, but whose age was less than 14 months, were coded as ‘unknown’

### Eligibility, receipt and retention of school and ANC-CWC nets

As shown in Table 4, one-fifth of households had a child in P2 or P6 at endline (18.8%); a similar percentage (19.3%) had had a child in P2 or P6 in the prior school year. Overall, 33.5% (29.2–38.1) of households had a child in P2 or P6 at either the 1st or 2nd round of school distribution. In the second round of school distribution in October 2014, however, eligible classes were changed to P4 and P5; the endline questionnaire was not updated reflect this. As a result, the endline does not accurately capture the proportion of eligible households in 2013–2014 that received school ITNs. Of these households, 37.5% (95% CI 31.8–43.7) reported receiving an ITN from the school distribution. Almost four percent (3.9%) of households had a currently pregnant woman; 28.5% (95% CI 15.0–47.5) of these reported owning an ITN from ANC. Of households with any children ages 12–35 months—the age group that would have been eligible for the measles booster during the entire pilot period—20.2% (95% CI 14.0–28.4) reported receiving an ITN from CWC. The proportion of households that owned at least 1 ITN from these channels was slightly lower than the proportion receiving ITN, suggesting some loss of these ITN due to wear and tear, or through redistribution to family members.

**Table 4:** Household eligibility for school, ANC, and CWC distribution during the pilot

	% of households with eligible individual at endline	% of households with eligible individual that reported receiving corresponding ITN	% of households with eligible individual that own at least 1 corresponding ITN
Schoolchild in P2 or P6 in 2012–2013	18.8	49.1 (40.6–57.7)	37.9 (30.6–45.7)
Schoolchild in P2 or P6 in 2013–2014	19.3	28.9 (22.1–36.8)	23.7 (17.5–31.3)
Schoolchild in P2 or P6 in 2012–2013 or in 2013–2014	33.5	37.5 (31.8–43.7)	28.9 (23.8–34.6)
Currently pregnant woman or child under 12 months	16.2	24.8 (17.6–33.8)	17.2 (11.7–24.6)
Child 12–35 months	20.4	20.2 (14.0–28.4)	10.4 (6.4–16.4)

Rates are only for ITNs reported as obtained during the pilot period

### Equity

**Fig. 4:**
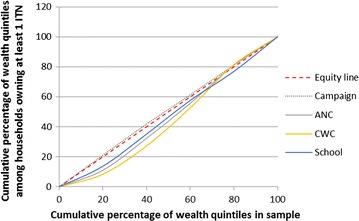
Lorenz concentration curves for mass campaign, ANC, CWC, and school ITNs, among households with at least 1 ITN

Figure 4 shows the Lorenz concentration curve, which demonstrates the equity in ITN ownership among households that received an ITN from the mass campaign, ANC, CWC, and schools. The mass campaign was very equitable, with a very slight pro-poor tendency (concentration index − 0.02; 95% CI − 0.04 to 0.00), as was ANC (concentration index 0.08; 95% CI − 0.02 to 0.18), CWC (concentration index 0.12; 95% CI 0.00–0.24), and schools (concentration index 0.07; 95% CI − 0.01 to 0.14). The overall concentration index score for any CD channel was slightly but significantly positive (ANC, CWC or school: concentration index 0.10; 95% CI (0.04–0.15)), indicating that although the continuous distribution channels were equitable as a whole, they were slightly more likely to be accessed by households from wealthier quintiles.

### Seasonal ITN use

**Fig. 5:**
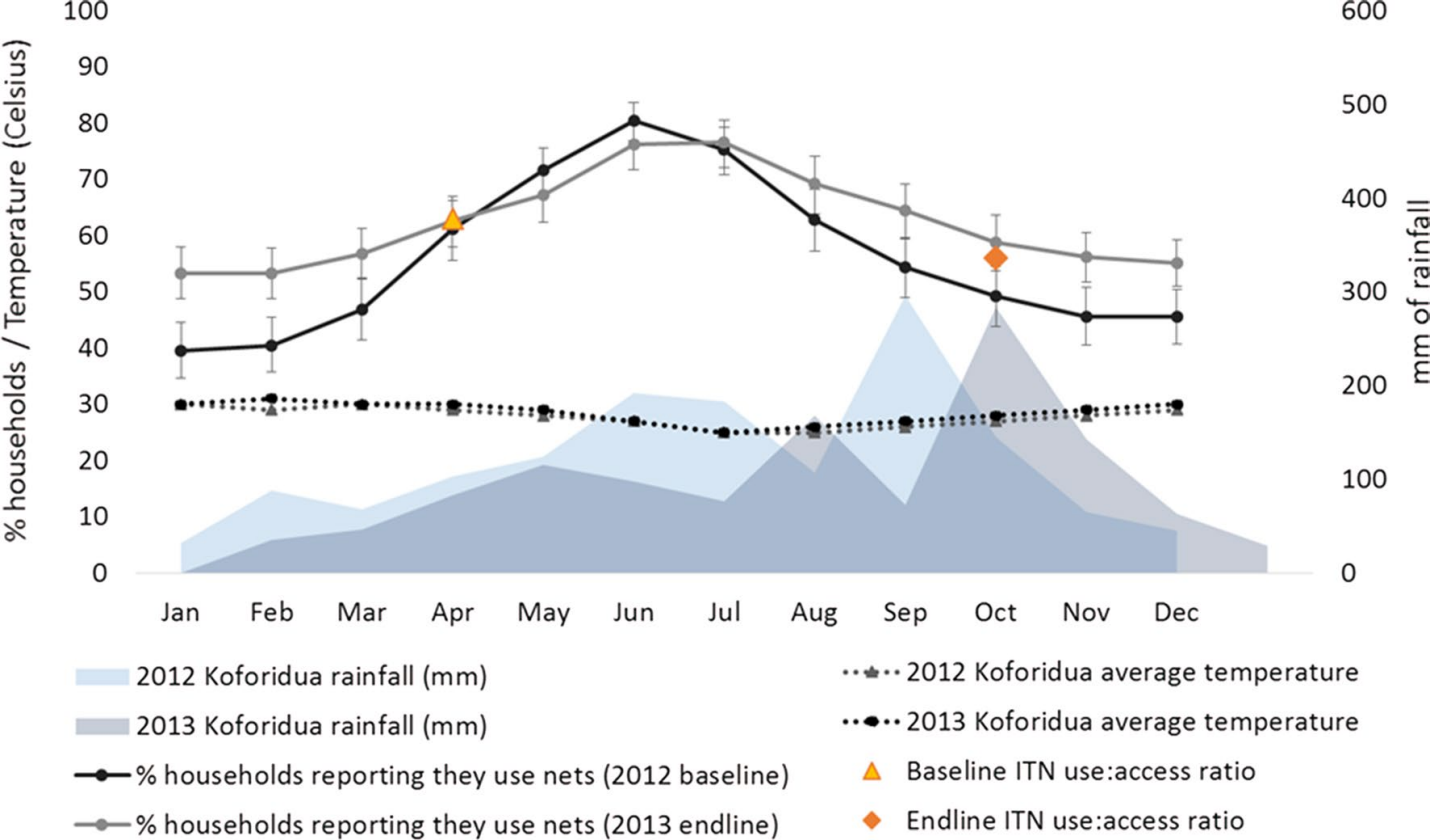
Monthly reported ITN use against average monthly temperature and rainfall

Respondents were asked which months of the year they generally use nets. Responses at baseline and endline are shown in Fig. 5 plotted against weather station data for Eastern Region’s capital city, Koforidua, from 2013 [25]. Net use increases in April as rains begin, peaking in June–July, and declining again in August–September during a brief dry/cooler season. Temperature does not fluctuate significantly during the year, but peak rains appear to correspond with declining seasonal net use. Differences in urban and rural seasonal net use patterns were assessed but not found to be statistically significant at either baseline or endline.

## Discussion

While there is a significant amount of literature on the effectiveness of mass campaigns, the literature on the effectiveness of continuous distribution strategies is still sparse. The results from this study suggest that the combination of ANC, EPI, and school distribution in two primary school classes did prevent some loss of ITN ownership and access, but was not sufficient to sustain or increase coverage following the 2010–2012 mass campaign.

The decline in household universal coverage and population access can be attributed to four key factors. First, the 18–24 month delay in the start of implementation of the continuous distribution channels provided time for campaign ITNs to be lost due to wear and tear, as is commonly seen after mass campaigns [26]. The continuous distribution programme was supposed to start within 12 months the campaign and ITN quantities were planned with that in mind. Second, the full strategy as originally conceived was not implemented. The additional minor channels included in the projected coverage—secondary school students, retail sector, and measles booster outreach programmes—indicated that only two classes of school distribution would be needed. However, without these additional channels, distributing nets to only two classes per year of primary school, even combined with ANC and CWC distribution, did not provide sufficient quantities of ITNs to maintain targets. Other countries implementing school distribution of ITNs have targeted 4 or even up to 7 primary school classes, in combination with ANC and EPI channels [18]. Third, given that less than a third of pregnant women in the sample owned an ITN from ANC, there may have been stock outs or otherwise insufficient ITNs available at ANC and CWC, reducing the contributions of these channels; similar availability ratios of ANC and EPI ITNs among ANC attendees and infants have been observed across several other countries [27]. Fourth, with only half of households with eligible P2 and P6 children in 2012-2013 reporting owning an ITN from schools, it is possible that implementation of this channel was compromised, or that sources of ITNs were misclassified or not clearly known by respondents. Thus, it is important for programmes to start planning for continuous distribution during and immediately after mass campaigns with the intention of starting within 12 months. These findings also suggest that it is important to commit to a minimum number of channels and implement them fully in the selected geographic area, as attempting to roll-out CD in a gradual way can negatively impact coverage.

These results also indicate that programmes can take pre-emptive steps to prevent coverage gaps when delays are foreseen, or when originally planned channels are not implemented. School distribution offer a high level of flexibility since the number of classes receiving ITNs can be added or subtracted from year to year based on need. Increasing the number of eligible classes when the first year of implementation is delayed can bridge the gap between campaigns and continuous distribution without adding a significant burden to operations.

The ANC and CWC channels were selected on the premise that they would reach the groups most biologically vulnerable to malaria, while the school channel was selected because schools had a wide reach amongst communities and could also reach complementary demographic groups. The intention of the school distribution strategy in Ghana was to provide recurring opportunities for households to receive ITNs as children move through their primary school years. The additional channels in the strategy were selected to reach households without vulnerable groups or younger children. However, due to lack of human and financial resources, these were not implemented at all. Moreover, the 15-month implementation period does not fully capture the long-term impact of the distribution strategy. It is important to monitor coverage over time to measure the performance of continuous distribution strategies.

Although largely equitable, ownership of continuous distribution ITNs was slightly pro-rich. This is not unexpected, as utilization of education and health services is usually higher among wealthier households, and since schools and ANC/EPI distribution benefited complementary socio-economic groups. Efforts to improve equity in access to and use of these services will likely improve the equity in ownership of ITNs from CD.

The use of ITNs the previous night declined from baseline to endline, as did the ratio of those using an ITN to those with access to an ITN. The proportion of respondents reporting using nets during different months of the year also increased slightly from baseline to endline in the drier months of the year (November–February). These seasonal patterns of ITN use provide additional context for the shifts in ITN use the previous night as reported at baseline and endline.

A key limitation of the research was the survey error in assessing the proportion of households with eligible P4 and P5 students in the 2013–2014 school year, as these were the students receiving ITNs just prior to the endline survey. This prevents specific assessment of the reach of the school ITNs into these eligible households. However, the proportion of households with eligible students over the entire 2-year period is likely to be similar whether or not the students in the second year are in P2/P6 or P4/P5. About a third of all households had an eligible P2 or P6 child during the 2-year pilot period. As Ghana’s national strategy has been to implement school distribution to P2 and P6 in years in between mass campaigns, it is important to recognize that this approach reaches one-third of households during the 2 years between campaigns.

A second key limitation of the research is the possibility of recall and misclassification biases, particularly for the source of ITNs within the home. Nearly as many households owned school ITNs as owned ITNs from an unknown or discrepant source. It is possible that these ITNs of unknown origin were from the continuous channels, and this could explain why omitting only ITNs coded as schools or health facilities did not result in statistically significant difference from all ITNs in Fig. 3. Improvements to the wording of the source of net question may help to clarify this in future surveys.

## Conclusion

Thirty-one (31) months after the mass campaign, the fifteen (15) months of continuous distribution activities had maintained levels of household ownership at least one ITN, but household ownership of one ITN for every two people and population access to ITN had declined. Ownership and access were higher with the CD programme than without. However, the number of ITNs delivered via ANC, CWC and two primary school classes were insufficient to sustain target coverage levels. Programme managers considering a combination of channels need to carefully analyse how each channel will contribute to coverage, customize the approach to work for the local context, and monitor ITN coverage to make adjustments. Future programmes should choose the minimum number of channels for a strategy, then implement continuous distribution strategies fully (with no stock-outs) within 1 year after a campaign or widen eligibility criteria (such as increase the number of classes) during the first year of implementation to make up for programme delays.
